# Reduced egocentric bias when perspective-taking compared with working from rules

**DOI:** 10.1177/1747021820916707

**Published:** 2020-05-22

**Authors:** Steven Samuel, Anna Frohnwieser, Robert Lurz, Nicola S Clayton

**Affiliations:** 1 Department of Psychology, University of Cambridge, Cambridge, UK; 2 Department of Psychology, University of Essex, Wivenhoe, UK; 3 Brooklyn College, City University New York, Brooklyn, NY, USA

**Keywords:** Theory of mind, egocentric bias, perspective-taking, level 2 perspective-taking

## Abstract

Previous research has suggested that adults are sometimes egocentric, erroneously attributing their current beliefs, perspectives, and opinions to others. Interestingly, this egocentricity is sometimes stronger when perspective-taking than when working from functionally identical but non-perspectival rules. Much of our knowledge of egocentric bias comes from Level 1 perspective-taking (e.g., judging whether something is seen) and judgements made about narrated characters or avatars rather than truly social stimuli such as another person in the same room. We tested whether adults would be egocentric on a Level 2 perspective-taking task (judging how something appears), in which they were instructed to indicate on a continuous colour scale the colour of an object as seen through a filter. In our first experiment, we manipulated the participants’ knowledge of the object’s true colour. We also asked participants to judge either what the filtered colour looked like to themselves or to another person present in the room. We found participants’ judgements did not vary across conditions. In a second experiment, we instead manipulated how much participants knew about the object’s colour when it was filtered. We found that participants were biased towards the true colour of the object when making judgements about targets they could not see relative to targets they could, but that this bias disappeared when the instruction was to imagine what the object looked like to another person. We interpret these findings as indicative of reduced egocentricity when considering other people’s experiences of events relative to considering functionally identical but abstract rules.

## Introduction

Although at a theoretical level we are aware that other people can have different perspectives to our own, we often ascribe our own perspectives to others even when these particular ascriptions are unwarranted. For example, we sometimes find it hard to ignore what we can see when trying to understand what someone with a more limited visual perspective might be referring to (Apperly et al., 2010; Keysar et al., 2003; Samuel, Roehr-Brackin, et al., 2019); we sometimes imagine that our preferences and opinions are shared by more people than is objectively the case (e.g., Ross et al., 1977); and when we learn something new, we have trouble recalling our earlier ignorance and fail to appreciate that others might not know presently what we did not know previously (e.g., Bernstein et al., 2004; Hinds, 1999). This “egocentric bias,” sometimes referred to as the “curse of knowledge,” could have far-reaching implications for our ability to be objective about the world around us (Risen & Critcher, 2011), and for our attitudes towards and interactions with others (Birch & Bloom, 2004).

An interesting aspect of egocentric bias is that it has been shown to be modulated by the need to take another person’s perspective, a process believed to utilise “theory of mind” (sometimes “mentalising”), our ability to understand others’ mental states (Premack & Woodruff, 1978). At first sight this description of egocentricity may appear obvious, since being *ego*centric appears to necessitate the presence of another person’s perspective. However, egocentricity in its broadest sense concerns the intrusion of what we know when making judgements that require us to ignore this knowledge; this can be because we are asked to take the perspective of another person, but it can also be because we are asked to process what is essentially the same information couched in other, non-perspectival terms. In other words, we can be egocentric even when we are not thinking about others. For example, in a common perspective-taking paradigm known as the Director Task, participants with full visual access to an array of items in a grid are instructed to select items according to an avatar’s more limited perspective, such that the instruction to select “the top vase” might require the participant to select the second vase down if the avatar cannot see the top vase because of a barrier. Adults make egocentric errors both when the task is described this way *and* when the task is instead described by a functionally equivalent abstract rule, such as “do not select items that are in front of an occluder” (Apperly et al., 2010; Legg et al., 2017). Interestingly, more egocentric errors are usually found on this task in the perspective-taking condition than the rule-based one (though see below), leading some to argue that we are more prone to egocentric errors when we act in a social context (e.g., Apperly et al., 2010).

However, this idea that we are more egocentric when reasoning socially/perspectivally than from rules—which we here term the social egocentricity hypothesis——is not universally supported. For example, adults have been found to show equivalent egocentricity when asked to confirm how many dots on a wall an avatar sees or an arrow “sees” (Santiesteban et al., 2015). Adults also respond at similar speeds when judging the relative spatial positions of objects from the perspective of a doll or a camera (Aichhorn et al., 2006) and show a similar degree of bias towards an object’s true location when indicating where someone with a false belief about its location will look for it and where a film will falsely depict that object to be (Samuel et al., 2018). There are also cases in which making judgements based on others’ perspectives appears to *reduce* egocentricity relative to nonmental reasoning. For example, on a Director Task, participants have also been found to make perspective-based judgements *faster* than rule-based judgements, without any consequent difference in accuracy across the two conditions (Dumontheil et al., 2010), and adults have been shown to be both faster and more accurate when making judgements based on agents’ false beliefs than false but nonmental states such as notes (Cohen et al., 2015; see also Samuel, Durdevic, et al., 2019). Support for reduced egocentricity when reasoning socially would be consistent in spirit with research that posits specialised mechanisms or processes for theory of mind and social reasoning (Baron-Cohen, 1995; Sugiyama et al., 2002), as well as with the fluidity with which we organise our language online to reflect shared and privileged knowledge between communication partners (Clark & Brennan, 1991).

Interestingly, the research described above typically involves manipulating the context of a task rather than the answer that participants need to come to. In other words, the manipulations are essentially instructional rather than truly functional because, in reality, participant could opt to perform the task in any way they like. For example, instead of following a rule such as “ignore objects in front of occluders,” participants could imagine another agent on the other side of the grid and treat the task as social/perspectival. The same logic can of course be applied in reverse. The fact that different patterns of behaviour are found despite this apparently superficial change underlines the power of different formulations of instructions to promote different perspective-taking strategies, such that participants might even be made to perform suboptimally (e.g., Apperly et al., 2010; Presson, 1982; Samuel, Durdevic, et al., 2019; Wraga et al., 2000; Zacks & Tversky, 2005).

**Figure 1.**
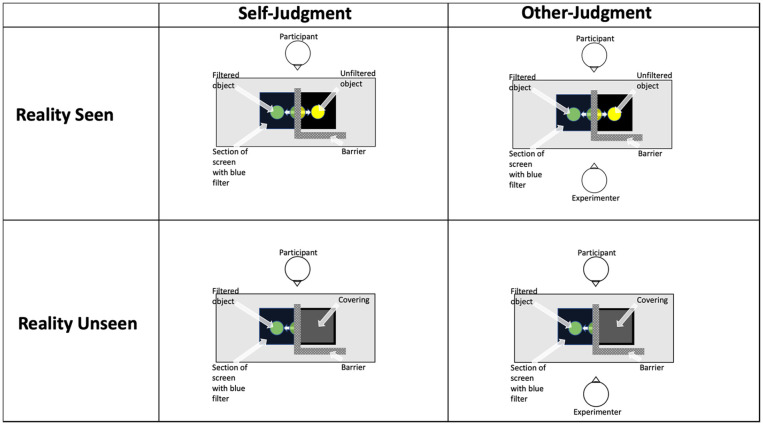
In Experiment 1, a blue transparent filter was placed on one half of the screen laid flat in front of the participant (and between the participant and the experimenter in the Other-Judgement condition). An L-shaped opaque barrier was placed over the unfiltered half of the screen. On each trial, the object passed under the barrier from the unfiltered to filtered section and back again before a colour scale appeared on the edge of the unfiltered side of the screen. In the Self-Judgement condition, the participant performed the task alone (the experimenter left the room) and in the Other-Judgement condition the experimenter sat opposite the participant and could only see the object through the filter. Participants were instructed to click on the scale according to either the colour the object appeared to them under the filter (Self-Judgement condition) or the colour the object appeared to the experimenter under the filter (Other-Judgement condition). In the Reality Seen condition, participants saw the full trajectory of the object prior to making their response. In the Reality Unseen condition, a covering/occluder was placed over the unfiltered section of screen, and thus the participant only saw the object through the filter.

In Experiment 1, we utilised this ability to elicit different strategies through instruction to test the social egocentricity hypothesis, which holds that egocentric errors increase under social perspective-taking demands. To do so, we gave participants a task that they could solve either with or without perspective-taking. The aim was to test whether simply changing the context of a task from a social to a nonsocial one would elicit an adjustment in the degree of egocentricity. Throughout the task, participants saw a series of coloured discs on a screen pass under a blue colour filter (see Figure 1). The filter altered the object’s colour, such that yellow would appear green, and green a bluish green. They were instructed to click on a colour scale to indicate the *filtered* colour, never the true colour. Participants always saw this filtered colour on every trial. We manipulated two variables. First, on one block, participants were shown the true colour of the object before and after it moved under the filter (the Reality Seen condition), and on another they were not (Reality Unseen). This manipulated the salience of participants’ knowledge of the true colour of the object. We reasoned that being shown the true colour would increase the likelihood of egocentric intrusion, “pulling” responses closer to the yellow end of the colour response scale. Note that the participant could ignore this information and instead focus on the filtered colour alone if they wished to. In this sense, the manipulation is primarily contextual.

Second, we manipulated the social context of the task. The social egocentricity hypothesis predicts that couching the task as a social, perspectival one increases egocentricity relative to couching the task as a simple perception task. To test this, participants were randomly assigned to one of two groups. In the Self-Judgement group, participants performed the task alone in the room and the instruction was always to indicate the colour that the participant herself had just seen through the filter. In the Other-Judgement group, the instruction was always to indicate which colour the *experimenter—*present throughout both blocks of the task*—* had just seen through the filter. Note that this change was again entirely contextual—participants could always rely on what they themselves had just seen if they chose to do so.

Our design was also aimed at tackling two important issues in some of the perspective-taking literature to date. First, we cannot always be certain that participants’ behaviour in the ostensibly “social” contexts is necessarily being guided by an understanding of another’s perspective. That is, although investigating participants’ perspective-taking behaviour in the context of avatars, dolls, and such provides an insight into strategies by which we *might* take actual people’s perspectives in real life, experimental conditions such as these may not represent the *typical* way in which we do so. There is a great deal of evidence to suggest that adults behave qualitatively differently in the presence of an actual human agent (i.e., with the potential for social interaction) rather than when faced with a depiction or simulation of one, such as avoiding eye contact with real people but making eye contact with depictions of them on a screen (see Skarratt et al., 2012, for a review). It could be that the salience of our egocentric viewpoint is modulated by precisely how social a supposedly social stimulus (in a psychology experiment) might actually be perceived to be. Given that reasoning about other people around us is the most naturalistic case, we investigated the social egocentricity hypothesis with a task in which participants were instructed to perform the task by judging how an object appeared either to themselves or to another person in the room.

A second issue concerns the *type* of perspective that participants are instructed to take. An important distinction has been drawn between understanding *whether* someone perceives something (i.e., Level 1 perspective-taking) and understanding *how* things appear (Level 2 perspective-taking; e.g., how a 6 can appear to be a 9 depending on where you are in relation to it; Flavell et al., 1981; Masangkay et al., 1974). Level 2 perspective-taking is usually regarded as a better test of the ability to represent other people’s mental states, since understanding how something appears is more likely to require an understanding of another’s actual experience. In contrast, understanding whether something is perceived is a computation that can be achieved through lower level processes such as reading someone’s external behaviour and reasoning geometrically or spatially (Heyes, 2014; Michelon & Zacks, 2006). In our experiment, the task was not to judge whether something was or was not visible to the other agent (it always was), but rather to make a judgement based on object appearance.

In sum, we hypothesised that participants would indicate colours closer to the object’s true colour when they were shown the object unfiltered just before they made their response (Reality Seen) compared with when the true colour was hidden on that trial (Reality Unseen). Given the discrepancies in the literature, we were more open-minded as to the direction of any effect of perspective-taking on egocentricity. However, given that our experiment was primarily about visual perspective-taking, and experiments that have tended to show greater egocentric bias in social contexts have usually involved visual perspective-taking rather than, for example, belief reasoning, we tentatively hypothesised in line with the social egocentricity hypothesis that there would be greater egocentric bias in the Other-Judgement than Self-Judgement condition.

## Experiment 1

### Method

#### Participants

A total of 40 participants, all UK nationals, were recruited in exchange for financial compensation. All participants gave informed consent and the study was approved by the University of Cambridge Psychology research ethics committee (PRE.2015.085). None of the participants showed signs of colour-blindness when tested with the City University Colour Vision test (3rd Edition). The data from two participants were later removed for evidence of having ignored the filtered (target) colour on the scale throughout the experiment, instead consistently (and accurately) indicating the true colour of the object. The final sample was thus 38 participants (*M*_age_ = 21.7 years, range = 18–36, males = 13), 19 in the Self-Judgement group and 19 in the Other-Judgement group. Participants were debriefed following the experiment.

#### Materials and procedure

Participants performed a colour judgement task in which they had to indicate the colour of an object (a disc on a screen) as seen through a blue colour filter. Participants were randomly assigned to judgements about how they perceived the object (Self-Judgement condition) or how another perceived the object (Other-Judgement condition). Within each group, the task varied based on whether participants were allowed to see the object’s true (i.e., unfiltered) colour immediately prior to making their response or not. Before the experiment participants were familiarised with the effect of a colour filter through observing videos of each of the 12 different-coloured disc used in the experiment moving behind the colour filter.

In the colour judgement task, the participant sat in a darkened room with a 24-in. screen laid flat (horizontally) in front of them. A blue transparent colour correction light gel filter covered one half of the screen (the half on the participant’s right—see Figure 1). In the Other Judgement condition, the other agent (the experimenter) sat opposite the participant but could only see the filtered section of the screen. Participants in the Other-Judgement condition were shown the agent’s restricted view prior to the experiment to make clear that only the participant could see the object unfiltered. In the Self-Judgement condition, participants performed the task alone. In both conditions, participants were instructed to watch videos in which a disc moved fluidly from the left unfiltered section to the right filtered section and back again. The disc spent an equivalent time on the left (5 s) and right side (5 s) of the screen during the videos.

All participants (whether in the self- or other-perspective groups) performed two blocks, order counterbalanced across participants. In the Reality Unseen block, the unfiltered section of the screen was covered so the participant only saw the object when it appeared through the filter. In the Reality Seen block, the participants saw the full trajectory of the object. Within each block 12 videos were repeated twice in randomised order to create 24 trials per block. Twelve 10-s videos were created for the experiment by generating a “yellow” object (RGB = 255, 242, 0) and “green” object (RGB = 0, 196, 100) and using a colour blender (www.meyerweb.com) to create 10 even steps between them (see Supplementary Material for full stimulus RGB coordinates). The discs therefore ranged from yellow to green in *real* colour, and hence from green to blueish-green when viewed through the blue filter used in the experiment.

At the end of each video and after the object had disappeared, participants were instructed to click on a response scale to indicate the colour that best represented either the colour of the circle as it had appeared to *them* through the colour filter (Self-Judgement) or how the circle would have appeared to *the experimenter*^1^ (Other-Judgement).

The response scale was always seen unfiltered. This judgement was made using a vertical 24-tile response scale that included 12 tiles reflecting the full range of the real colours of the discs in the experiment as well as an additional 12 tiles that were a continuation of these steps in colour towards the blue end of the colour spectrum (RGB = 0, 142, 199). These steps can be seen on the horizontal axis on the graphs in Figure 3. On half the trials the scale ran from yellow (top) to blue (bottom), on the other half the orientation of the scale was reversed. On each trial participant responses to the scale were recorded based on the vertical location (in pixels) that they had clicked on the scale.

### Results

Initial analyses found that the data from one variable (Reality Unseen in the Other-Judgement condition) deviated from normality (Shapiro–Wilk test, *p* = .047), and the others did not (*p*s > .2). A log transformation did not change this pattern of results. We therefore proceeded with parametric testing but cross-referenced these with parallel, nonparametric tests. The order in which participants performed the task (Reality Unseen first or Reality Seen first) had no significant effect on performance, either in terms of a main effect of Order or in interaction with this (all *p*s > .09). We thus collapsed over this factor in our main analyses.

**Figure 2.**
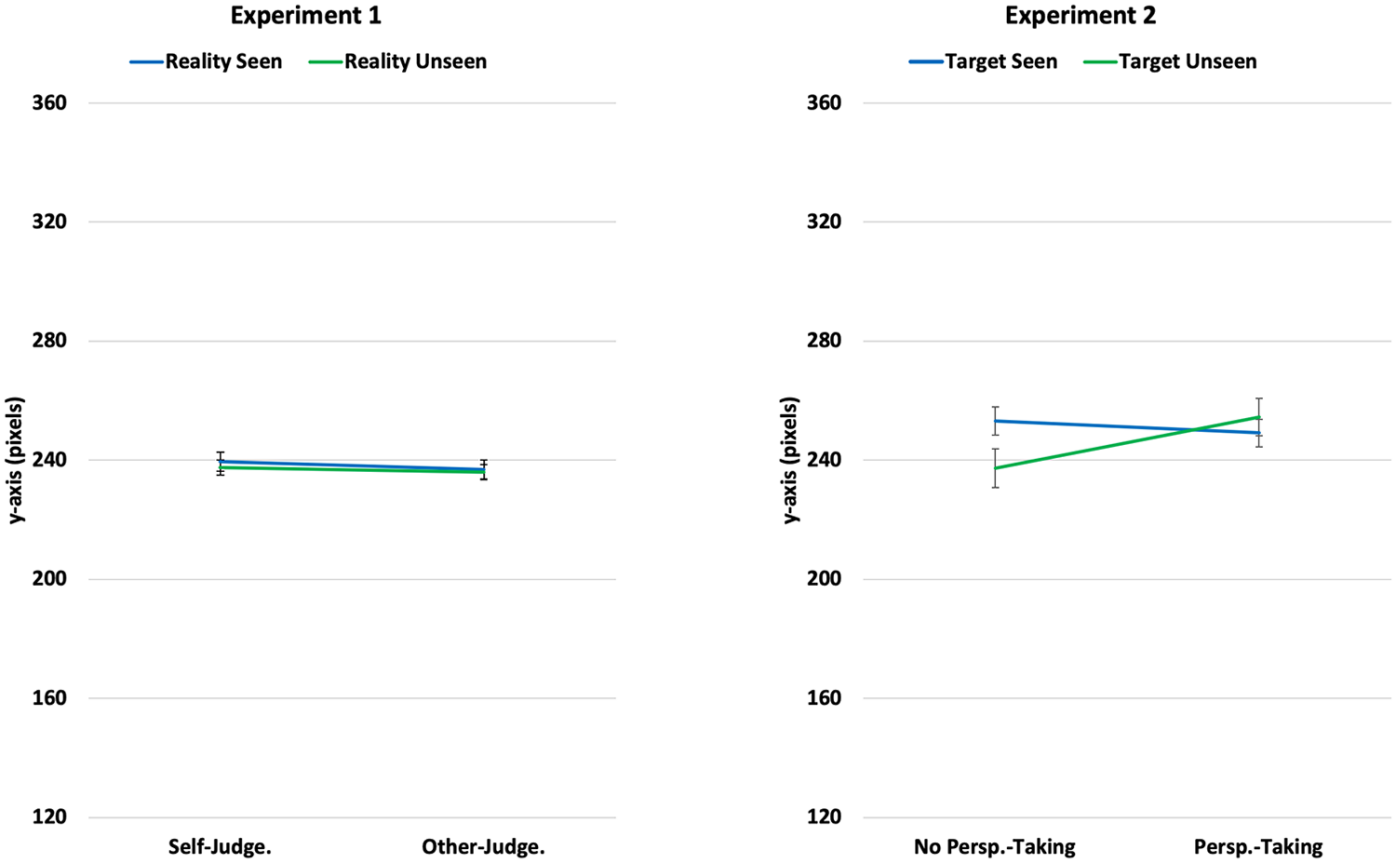
Condition means with standard error bars, for Experiments 1 (left panel) and 2 (right panel). Lower values on the scale indicate judgements closer to the true (unfiltered) stimulus colour, and hence a response closer to the actual colour of the object.

The condition means are displayed in Figure 2 (left panel). Participants in the Other-Judgement condition indicated points on the scale for the Reality Seen (*M* = 237, 95% confidence interval [CI] = [231, 243]) and Reality Unseen (*M* = 236, 95% CI = [231, 241]) blocks that were only one pixel apart. In the Self-Judgement condition, there was only a two-pixel difference between Reality Seen (*M* = 240, 95% CI = [233, 246]) and Reality Unseen (*M* = 238, 95% CI = [232, 243]).

**Figure 3.**
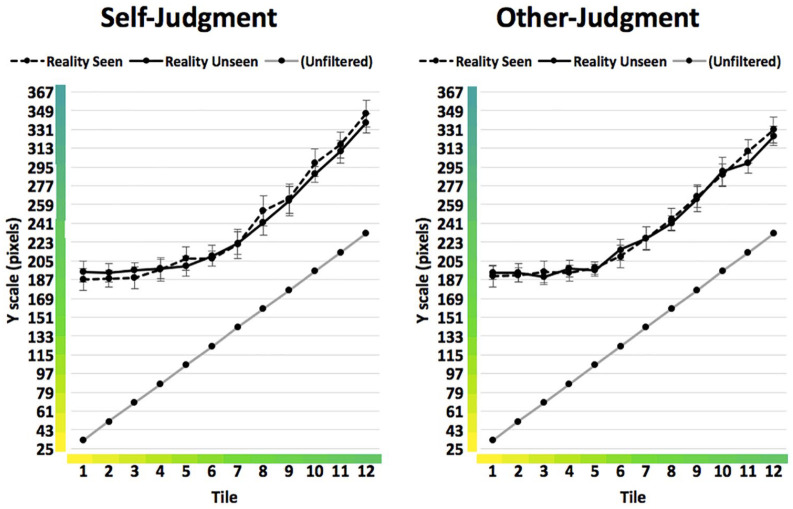
Condition means with 95% confidence intervals (Experiment 1). When unfiltered, Tile 1 represents a prototypical yellow and Tile 12 a prototypical green. Each horizontal line on the *Y* axis represents the border of a tile on the response scale.

The means broken down by each of the 12 tiles are displayed in Figure 3. Visual inspection of the results suggests the altering effect of the colour filter was strongest for yellows and weakest for greens, and that different types of yellow were perceived similarly when seen through the filter. Nevertheless, the graph gave no visual indication of any differences between conditions.

We conducted a 2: Condition (Other-Judgement vs. Self-Judgement) × 2: Knowledge (Reality Unseen vs. Reality Seen) mixed-design analysis of variance (ANOVA) with repeated measures over the last factor. The analysis found no main effect of Knowledge, *F*(1, 36) = 0.892, mean square of the error (*MSE*) = 44.216, *p* = .351, $\eta_{p}^{2}$ = .024, or Condition, *F*(1, 36) = 0.331, *MSE* = 264.668, *p* = .569, $\eta_{p}^{2}$ = .009, and no interaction, *F*(1, 36) = 0.106, *MSE* = 4.708, *p* = .746, $\eta_{p}^{2}$ = .003. The absence of a statistical difference between the two Knowledge conditions in the Other-Judgement condition was also supported by a nonparametric Wilcoxon signed-rank test, *U* = 86, *Z* = .362, *p* = .717. This highly consistent performance across both the Reality Seen and Reality Unseen conditions, coupled with the absence of any interaction with Condition, suggests consistency in participants’ judgements of the filtered objects’ colour regardless of whether they were reminded of its true colour or not, and regardless of whether they were making judgements from their own perspective or the perspective of another person.

Given the absence of any statistically significant effects, we conducted a test of the strength of the null hypotheses that (a) seeing the true colour of the object (Reality Seen) does not cause performance to vary relative to when the true colour of the object is occluded (Reality Unseen) and (b) taking another’s perspective (Other-Judgement condition) rather than one’s own (Self-Judgement condition) has no effect of performance. To do so, we ran a Bayesian analysis of the ANOVA (Condition × Knowledge). We adopted Dienes’ (2014) suggestion that meaningful support for a null result is data that is at least three times as likely under the null than alternative hypothesis. The analysis found that the absence of a main effect of Knowledge was *four* times more likely under the null (BF_10_ = 0.258), and the absence of a significant interaction with Condition was approximately *nine* times more likely under the null (BF_10_ = 0.117). In addition, the absence of a main effect of Condition was almost three times as likely under the null (BF_10_ = 0.364). In sum, both null hypotheses were supported.

### Discussion

In Experiment 1, we gave participants a task in which they were required to judge the apparent colour of an object as seen through a blue colour filter. On half of the trials, the participant saw the object’s true (unfiltered) colour immediately before responding, and on the other half they did not. In addition, half the participants were asked to make judgements about the object’s apparent colour from their own perspective, and the other half were asked to make the same judgements from the perspective of another person.

Contrary to both our predictions, we found no statistical difference in performance according to whether participants were reminded of the object’s unfiltered colour, or according to whether they took the other person’s perspective or their own. The consistency of responses across both groups and both conditions is particularly noteworthy. Across both the Self-Judgement/Other-Judgement and Reality Seen/Unseen comparisons, the mean response in one condition was always located within 16 pixels (the width of one tile) of the matched trial in the other condition. Together with the support for the null hypothesis from the Bayesian analyses, the evidence suggests that young adults’ judgements of the apparent colour of an object are not influenced by their own knowledge of that object’s true colour, nor by the instruction to take another’s perspective instead of their own. The social egocentricity hypothesis was not supported.

The finding that the manipulation of the salience of the object’s true colour did not interfere with performance is perhaps explained by the ability of participants to effectively ignore that information. In other words, participants may have simply restricted their attention to the target while it was filtered. The second outcome, concerning the absence of a difference between the social- and nonsocial contexts of the task, was more unexpected. Recall that previous research with (for example) the Director Task and false belief tasks had found systematic differences in performance simply by altering the context that the task was set in (Apperly et al., 2010; Cohen et al., 2015; Legg et al., 2017; Samuel, Durdevic, et al., 2019). For example, in the Director Task, the switch between social and rule-based performance could be the presence versus absence of a humanoid avatar and the wording of instructions, just as in our study. In false belief tasks, the difference is often an even more limited adjustment, based exclusively on the text of the instruction. It may be that either one or both of (a) using a real human agent and/or (b) employing a Level 2 perspective-taking task means that participants perform the same way regardless of the social (or otherwise) context of the task. Alternatively, it could be that the task was not sensitive enough to detect any modulations of egocentricity, or the social context was made less social by the use of the experimenter rather than a truly naïve agent.

**Figure 4.**
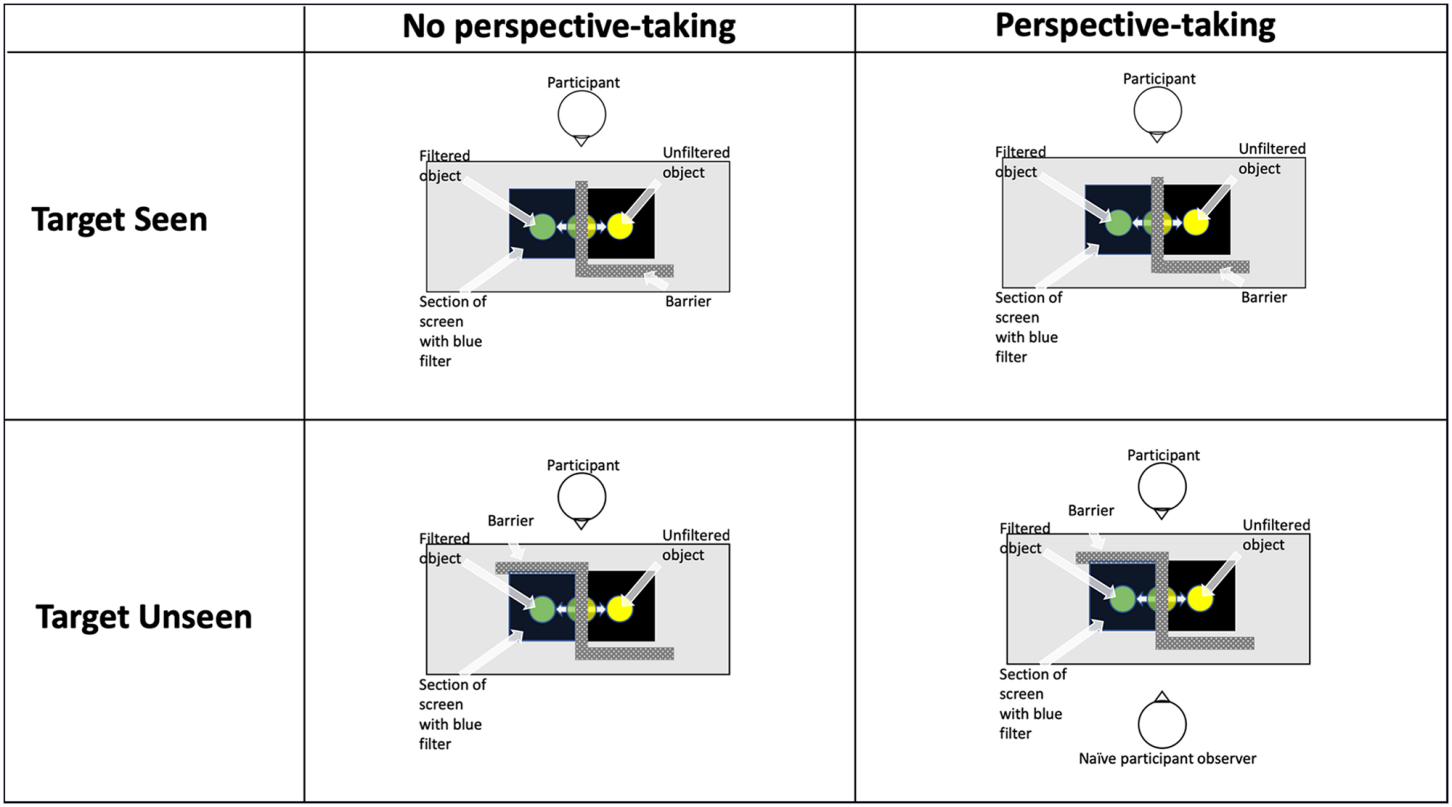
In Experiment 2, participants made judgements about targets they could see (Target Seen condition) or could not see (Target Unseen). Unlike Experiment 1, the object’s true colour was never hidden. Participants in the Perspective-Taking group were instructed to indicate what colour the other participant saw through the filter in the Target Unseen condition.

We conducted a second experiment to address these issues. In it we changed the design such that rather than hiding the true colour of the object on half the trials, we now hid the *target* (filtered) colour behind a barrier for half the task. This created a Target Seen block and Target Unseen block (see Figure 4), the order of which was counterbalanced such that half received the Target Seen block first. The object’s true colour was now always displayed immediately prior to participants’ responses (in a sense, the entire experiment was now in the “Reality Seen” context). If participants are biased by their knowledge of reality, then this bias should be more likely to intrude when judgements are being made under the increased level of uncertainty in the Target Unseen block. In this block, participants had no perceptual access to the filtered colour and therefore had to rely on their own understanding of how the filter had altered the object’s true colour, based on the same familiarisation phase at the beginning of the task as in Experiment 1. This new manipulation also allowed us to speak to a second and related concern, namely that we may not have found bias simply because the task was too easy. By removing from view the very target participants had to think about, it was now impossible in the Target Unseen block to rely on one’s recent perceptual experience.

As in Experiment 1, one half of the participants performed the task alone and were instructed to make judgements based on their own reasoning. The other half of the participants performed the Target Unseen block with another agent and were instructed to indicate the colour that agent saw through the filter. Note that this time the participant herself did not see the colour through the filter on these trials, only the other agent. This time, instead of the experimenter we recruited a second and naïve participant to be the observer. Although the issue of observer naivety is usually considered important for tasks in which a confederate is engaged in language-use with a naïve participant (Kuhlen & Brennan, 2013), some theories of egocentric biases posit that they might arise only when considering a more naïve or ignorant other (Birch & Bloom, 2004). This manipulation eliminated the possibility that participants did not show greater bias in the social context simply because of the type of agent they were reasoning about.

Finally, we preregistered our methods and analyses for Experiment 2: https://osf.io/65dsb/register/5,771ca429ad5a1020de2872e. Our primary hypothesis was again in line with the social egocentricity hypothesis, namely that participants should indicate colours closer to the object’s true colour when asked to take the perspective of a naïve agent; in other words, a social context would promote more egocentric responses. We set as evidence for this hypothesis a statistically significant interaction between Group (Perspective Taking vs. No Perspective-Taking)^2^ and Target (Target Seen vs. Target Unseen), favouring judgements closer to the true colour in the Target Unseen, Perspective-Taking condition than in the Target Unseen, No Perspective-Taking condition. If an interaction should not be found, then there should at least be a main effect of Target, such that participants should indicate colours closer to the true colour when the target was hidden by a barrier compared with when it was visible. This would indicate that the task was sensitive enough to elicit modulations in egocentricity, should no evidence of any effect of perspective-taking be found.

## Experiment 2

### Method

Except where indicated, the procedure for Experiment 2 was the same as for Experiment 1.

#### Participants

A power analysis using G*Power indicated that two groups of 23 participants were required for an 90% chance to detect an interaction of medium effect size, assuming a correlation between variables of .5. Eligibility requirements were the same as for Experiment 1. The No Perspective-Taking group consisted of 22^3^ individuals (*M*_age_ = 24.6 years, range = 18–35, males = 6), and the Perspective-Taking group consisted of 23 individuals (*M*_age_ = 21.9 years, range = 18–30, males = 6, nonbinary = 1). We also recruited 23 additional (British) participants to act as observers only (*M*_age_ = 24 years, range = 18–41, males = 5). In all other respects, Experiment 2 was identical to Experiment 1.

#### Materials and procedure

All participants performed one block in which the target (the filtered object that they were making judgements about) was visible (Target Seen), and one block when it was occluded (Target Unseen). As before, block order was counterbalanced across participants such that half performed the Target Seen block first. Participants in the No Perspective-Taking group were instructed to indicate on the same scale as before the colour of the object through the filter, on both blocks. The participants in the Perspective-Taking group performed the Target Unseen condition differently. For this block, a second (observer) participant entered the lab and was instructed to watch the object when it was visible (i.e., when it was filtered). The main participant was instructed to indicate the colour of the object that the other person saw. The observer participant was also a British national and had not met the main participant prior to the task.

As in Experiment 1, participants watched each of the 12 videos (one per colour) once with full visibility prior to starting the first experimental block. This was necessary for participants to see how the filter altered the colour of the object. The observer participants were never present during this phase and never saw the disc except when it was filtered.

### Results

The condition means are displayed in Figure 2 (right panel). Data were normally distributed, and we therefore proceeded with a 2: Group (No Perspective-Taking vs. Perspective-Taking) × 2: Target (Target Seen vs. Target Unseen) mixed-design ANOVA. The order in which participants performed the task (Target Unseen first or Target Unseen first) showed an influence on performance, with responses on average 14 pixels closer to yellow across the task as a whole if the Target Seen block was performed first (*M* = 242) than second (*M* = 256; *p* = .029). Crucially however the factor Order did not interact with either the factor Target (Target Seen vs. Target Unseen), or the factor Group (No perspective-Taking vs. Perspective-Taking), nor was there any three-way interaction (all *p*s > .13). We thus collapsed over this factor in our main analyses.

The analysis yielded no main effect of Target, *F*(1, 43) = 1.368, *MSE* = 448.655, *p* = .249, $\eta_{p}^{2}$ = .031; participants were not more likely to judge hidden targets as closer to the object’s real colour (*M* = 246, 95% CI = [237, 255]) than visible targets (*M* = 251, 95% CI = [244, 258]). There was also no main effect of Group, *F*(1, 43) = 0.988, *MSE* = 976.372, *p* = .326, $\eta_{p}^{2}$ = .022; participants in the Perspective-Taking group were not more likely to judge occluded targets as closer to the object’s real colour (*M* = 252, 95% CI = [243, 261]) than participants in the No Perspective-Taking group (*M* = 245, 95% CI = [236, 255]). However, as we had hypothesised there was indeed a significant interaction, *F*(1, 43) = 5.624, *MSE* = 448.655, *p* = .022, $\eta_{p}^{2}$ = .116, but it was not in the expected direction. Instead, the interaction suggested that judgements were closer to the object’s true colour in the No Perspective-Taking group in the Target Unseen condition. In other words, participants were *less* egocentric when judging the colour the other agent saw relative to simply imagining for themselves what the hidden filtered colour was.

We examined this interaction by means of two post hoc paired sample *t* tests (Bonferroni-corrected), comparing judgements in the Target Seen and Target Unseen condition. Participants in the No Perspective-Taking group indicated judgements closer to the object’s true colour when the target was occluded than when it was visible, with a medium effect size, *M*_Diff_ = 16, 95% CI = [4, 28], *t*(21) = 2.724, adjusted *p* = .026, *d* = 0.581, but participants in the Perspective-Taking group did not, *M*_Diff_ = –5, 95% CI = [–19, 9], *t*(22) = 0.796, adjusted *p* = .870, *d* = 0.166. In this latter group, Bayesian analyses found that the data were 7.5 times more likely under the null that participants did not indicate colours closer to the true colour when perspective-taking than when not (BF_10_ = 0.132). In contrast, the statistically significant effect in the No Perspective-Taking group was eight times more likely under the *alternative* than the null (BF_10_ = 8.073).

**Figure 5.**
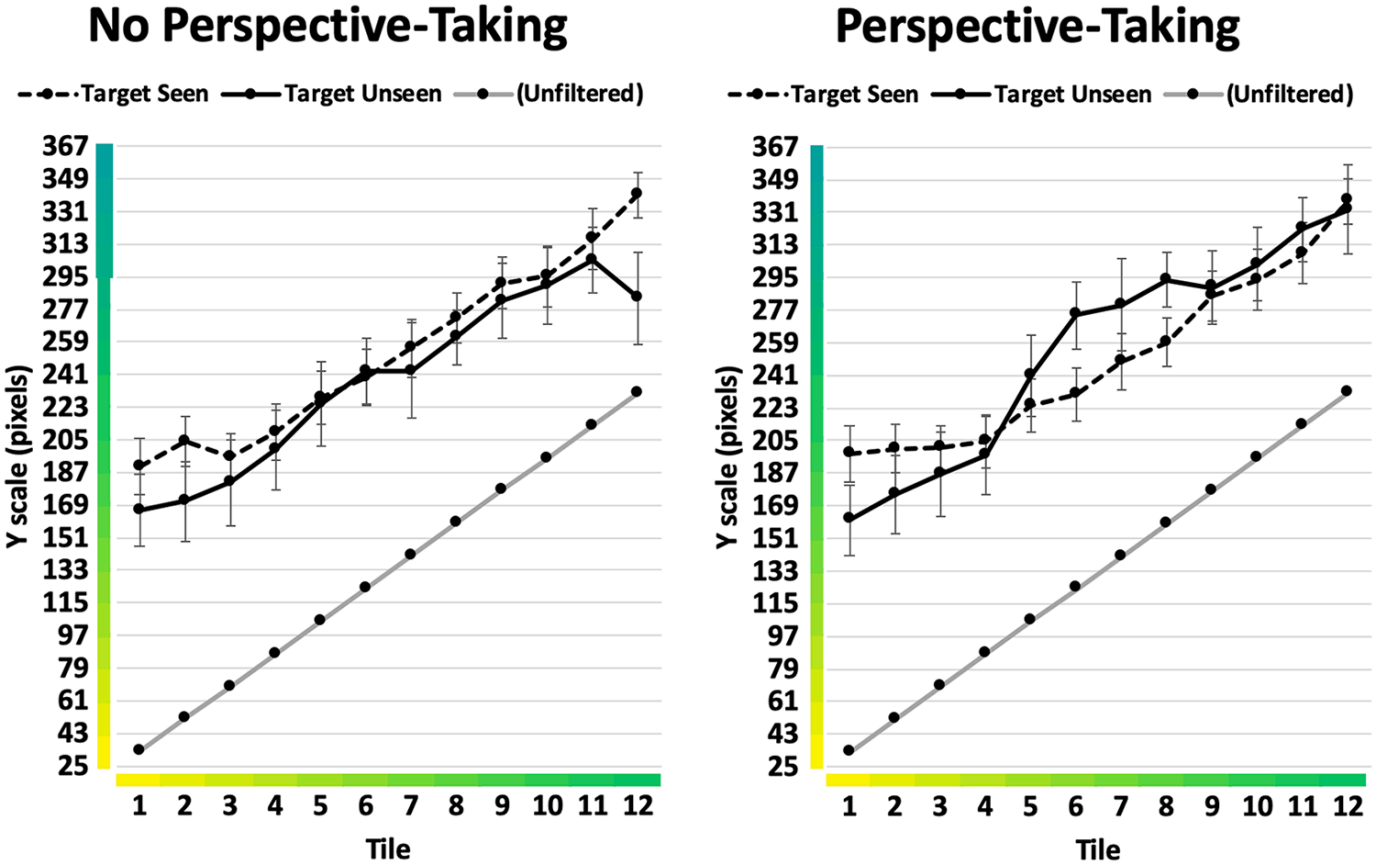
Condition means with 95% confidence intervals (Experiment 2). When unfiltered, Tile 1 represents a prototypical yellow and Tile 12 a prototypical green. Each horizontal line on the *Y* axis represents the border of a tile on the response scale.

The means broken down by each of the 12 tiles are displayed in Figure 5. Looking at these means individually, participants in the Perspective-Taking group indicated colours closer to the true object colour in the Target Unseen than in the Target Seen conditions for five of the 12 colours. These were the four yellowest colours and the greenest colour. In contrast, in the No Perspective-Taking group, bias towards the true colour was greater for 11 out of the 12 colours (the sole exception being the sixth colour on the scale, approximately half-way between yellow and green). This pattern suggested that the finding of greater bias in the No Perspective-Taking group was not the result of a minority of outlying data points but rather an almost 100% consistent pattern.

### Discussion

In Experiment 2, we hypothesised (again, in line with the social egocentricity hypothesis) that participants would be more egocentric when perspective-taking than when working out for themselves what the hidden colour would be. This should have been indexed by an interaction, finding judgements closer to the true colour of the object in the Target Unseen, Perspective-Taking condition than in the Target Unseen, No Perspective-Taking condition. What we found, however, was an interaction and subsequent follow-up tests that suggested the opposite; employing a barrier to hide the target colour from the participant did increase egocentricity but *only when participants performed alone*. In sum, our results not only failed to support the social egocentricity hypothesis, they patterned in the reverse; participants showed evidence of resistance to egocentric bias when imagining another’s perspective.

Overall, the results of Experiment 2 suggest an important difference in the degree of egocentric intrusion when making judgements that are *social* or *perspectival* in their format relative to judgements that are essentially about logical reasoning (working out what a filter has done to a colour): imagining what another person sees appears to have the effect of helping overcome egocentricity.

## General discussion

Over two experiments we gave participants a task in which they were required to ignore an object’s true colour and instead judge its apparent colour as seen through a blue colour filter. In Experiment 1, half the participants were asked to make judgements from their own perspective, and the other half were asked to make judgements from the perspective of another person in the room. We hypothesised that participants would be more likely to indicate colours closer to the object’s true, unfiltered colour when they were reminded of this colour just prior to responding. We found no evidence to support this hypothesis; mean judgements were approximately equivalent across both conditions. We also hypothesised—in accordance with the social egocentricity hypothesis—that participants might indicate colours closer to the true colour of the object when they were asked to take another person’s perspective instead of their own. Again, we found no evidence that participants’ judgements varied according to the type of instruction they received. In Experiment 2, a new group of participants made judgements about the same stimuli, but this time the target object was sometimes occluded, meaning participants had to infer the filtered colour from their view of its true colour. In addition, instead of making judgements about what the experimenter saw, a naïve second participant was introduced for the perspective-taking block of trials. We reasoned these manipulations would provide more fertile ground for egocentric biases to arise. Those participants who performed the task on their own showed an increase in egocentricity when the target was hidden compared with when it was visible. However, participants who were asked to take another’s participant’s perspective showed no such difference. This outcome was contrary to our tentative hypothesis that egocentric bias would increase when perspective-taking.

Overall, across two experiments participants did not show any difference in their ability to ignore an object’s true colour when making a judgement about its filtered colour, so long as those judgements were couched in a social, perspective-taking context. In the first experiment, we cannot be certain that this was not due to the simplicity of the task creating a ceiling effect, whereby participants ignored the true colour of the object and responded based on what they themselves saw throughout. However, in the second experiment we can rule out this possibility because egocentricity did vary in the nonsocial version of the task once we blocked the target object from view. The difference in performance was not trivial (a medium effect size according to Cohen’s conventions). Taken together, we interpret this finding as suggesting that making appearance judgements about objects can elicit stronger egocentric biases under particularly demanding conditions (such as when the object being judged is not visually accessible), but that performing the task *perspectivally* rather than non-perspectivally serves to reduce or eliminate this added difficulty.

Our findings therefore appear more consistent with theories that treat reasoning about others’ mental states as the outcomes of a specialised cognitive mechanism for social reasoning (Baron-Cohen, 1995; Sugiyama et al., 2002), or research that suggests more efficient processing of social than nonsocial representations (Cohen et al., 2015; Samuel, Durdevic, et al., 2019). We do not claim, however, that our results provide direct evidence for either. It is equally likely, for example, that we are less biased when perspective-taking because we are simply more practiced at imagining what others see rather than what colour filters do to objects. Instead, we prefer a more conservative interpretation, namely that when more than one strategy is available when making judgements about visual appearances, we are less susceptible to egocentric intrusion when we adopt a perspective-taking strategy.

Our findings offer a potential explanation for the fluctuations in egocentricity in other tasks in the literature. Recall that previous research using the Director Task—also a visual perspective-taking task—has usually supported the possibility that social reasoning should *increase* egocentricity relative to following functionally identical but non-perspectival rules (Apperly et al., 2010; Legg et al., 2017). Our results found the opposite. There are a number of possible reasons why this might be the case, because our design differed from the Director Task in a number of important ways, and any one or combination of these might have resulted in this reverse pattern. To take only three examples: (a) we used a real human agent instead of an avatar, (b) our task did not involve locating targets referred to linguistically, and (c) participants were only ever presented with a single object at a time rather than an array. As there are not any empirically established reasons to believe that these contrasts necessarily modulate egocentric biases in any reliable manner or any particular direction, it would be interesting for future research to begin to tackle precisely these questions so that we might be able to develop clearer explanations for differing patterns of egocentric bias across different methodologies. Given the present paucity of such data and the novelty of our own methodology, we do not see our findings as contradicting outcomes from paradigms such as these owing to the clear differences between them.

We also cannot from our data draw any direct comparisons between performance with real human agents and simulations or depictions of agents such as avatars and dolls, and so we are only capable of speculating upon how the type of agent might influence performance on the comparison of a real human agent versus no agent at all. We might speculate, for example, that reasoning about other people reduces bias while reasoning about *simulations* of people or nonhuman entities might sometimes *increase* it. This could explain why egocentricity tends to rise or efficiency decrease when we make judgements about nonmental representations such as photos, notes, or maps, or base our judgements on avatars rather than people. Such an argument clearly has an intuitive appeal, as it would appear unlikely that humans should have evolved to have the greatest difficulty in reducing egocentricity when working in the most “naturalistic” context. However, it is important to highlight that the wider literature on perspective-taking does not support a simple linear pattern of egocentricity reducing to zero the more “human” the other agent is. For example, perspective-taking with real human others certainly does not guarantee the elimination of egocentricity (Keysar et al., 2003; Mozuraitis et al., 2015, 2018; Wardlow, 2013; Wu & Keysar, 2007), and as described earlier, similar performance has been found when participants were asked to take the visual perspectives of dolls and inanimate objects such as cameras (Aichhorn et al., 2006) and even asterisks (Michelon & Zacks, 2006). These findings are spread over a number of different task types and methodologies, and an interesting question for future research would be to attempt to disentangle the various effects of the type of agent being reasoned about (human through to inanimate object), the form of the perspective (Level 1 vs. Level 2), and the task demands (e.g., primarily visual or primarily referential) to try to develop a more general account of egocentricity. Regardless of the accuracy or otherwise of such speculations (which only further research can be in a position to judge), we were specifically concerned with the processing of other *people’s* perspectives, and in that sense we feel our paradigm speaks to the particular issue of social/perspectival versus asocial/rule-based reasoning. In sum, our data suggest that when making judgements about the visual appearance of an object, a pattern of reduced egocentricity might occur when participants are performing the task as a perspective-taking task about a real and present human other relative to an asocial and non-perspectival context.

It is important to highlight the similarities and differences between our task and other tasks that look at egocentric biases and perspective-taking. One anonymous reviewer pointed out that at no point in our study was there a conflict between what the participant’s response should be and what the other agent’s response should be. For example, in the Director Task, the instruction to select the “top cup” will, on critical trials, require a different response depending on viewpoint. A self-perspective response when the task was to take the other perspective is a clear indication of a failure to take perspectives and a reliance instead on what the participant herself sees. In our study, the correct answer was always the same filtered colour regardless of perspective, and as a result we may not know which perspective participants made responses from, with consequences for our interpretations of egocentric bias. We agree with this characterisation of our study up to a point—there was no conflict between self and other perspectives in our study, which is unorthodox. However, this does not mean that participants could not demonstrate egocentricity. Overall, our measure of egocentricity came not from a privileged perspective vis-à-vis another agent, but rather privileged knowledge about the object that was the focus of the task. The crucial question concerned whether such bias would vary as a function of the way the task was framed.

Another point raised by an anonymous reviewer concerned whether the mere presence of the other agent might have elicited a different strategy in participants, independently of the instruction to take that agent’s perspective. There was no evidence, however, that responses changed as a function of either the presence of an agent *or* the instruction to take that agent’s perspective. This is indicated by the absence of a main effect of or interaction with Group in Experiment 1, and the absence of an effect of condition in the Perspective-Taking group in Experiment 2. Moreover, given that we explicitly instructed participants to take the agent’s perspective, not just to remain aware that there was another agent present in the room, and that the agent could not observe the participant’s responses on the colour scale in any case, it is to our minds more likely that the attenuation of egocentric bias in Experiment 2 was due to perspective-taking specifically. Nevertheless, it is very difficult with our data to separate any effect of the agent’s presence from the instruction to take that agent’s perspective, as the two always co-occurred, and we cannot definitively rule this possibility out.

The contrast between performance while perspective-taking relative to performance while not perspective-taking is also relatively uncommon in the literature, but was the principal focus of the present research. This contrast is not the same as comparing perspective-taking with avatars and nonsocial but still essentially *perspective-based* control stimuli like arrows or cameras, because reasoning about the effect of a filter has an abstract and spatially “adirectional” quality that is not shared by such stimuli. In our view, our task therefore has no true analogue in the literature, but is probably most closely aligned with those versions of the Director Task which have contrasted perspective-taking with an avatar and a rule-based control (Apperly et al., 2010; Dumontheil et al., 2010; Legg et al., 2017). This is in no way a comment on the value or validity of other research in the field, but simply a necessary caveat to any interpretations of support or contradiction when our results are considered in the light of work focussing on Level 1 perspective-taking (Samson et al., 2010), embodied perspective-taking (Surtees et al., 2013b), or *altercentric* rather than egocentric interference (Elekes et al., 2016; Samson et al., 2010; Surtees et al., 2016), or in the light of performance in studies with depictions of agents rather than real human agents (Surtees et al., 2013a, 2016).

Our results *do*, however, corroborate previous research suggesting that participants can be made to engage two different strategies—one perspectival and one not—to arrive at what should be the same conclusion, with different behavioural outcomes as a consequence. Certainly this was the case in Experiment 2. In Experiment 1, the task may simply have been too easy for strategy changes to make any difference. Overall, this pattern of experimentally manipulated strategy choice is an interesting one for psychological research more generally, because it points to the ability of participants to select a less efficient or less accurate strategy among the options available to them simply as a result of prompting. Of course, it is very possible that participants do not *know* they have selected the more biased route to an answer in our study, as the difference might be too small to detect at a conscious level. An interesting question for future research would be to look for this moment of conscious awareness; how strong does our egocentric bias need to be before we become consciously aware of it?

There are two further points of interest from our results that we feel deserve mention. First, it is important to make clear that we do not claim that participants in our study showed *no* egocentric bias at any time. Our design did not allow the possibility to measure participants’ accuracy of judgement because the colours seen through the filter were not created digitally, and we did not measure these colours. A suggestion for future research would be to utilise colour measurement technology to measure participants “absolute” accuracy in their colour judgements. For our purposes, it was enough to test for differences in the direction of judgements on the colour scale to establish the impact or otherwise of varying conditions in the task. We therefore interpret our results in relative rather than absolute terms, namely that increasing uncertainty increases egocentricity, and perspective-taking appears to eliminate this increase.

Second, we found an irregular pattern of judgements through the 12 colour tiles in Experiment 2, when the target was occluded. These showed that participants from both the No Perspective-Taking and Perspective-Taking group indicated judgements closer to the true colour of the object when that colour was *yellow* (Tiles 1 and 2), but not when it was green. In fact, performance on green tiles varied somewhat according to group, with the No Perspective-Taking group displaying a fairly even incline in responses through the green tiles, whereas the Perspective-Taking group displayed a more pronounced shift close to what might be considered the yellow–green category boundary. This shift appeared to not only to reduce bias but even to reverse it, because for some discs participants indicated judgements further from the true colour when perspective-taking than when not. One explanation, albeit speculative, is that participants in the Perspective-Taking condition made greater use of colour labels to make their judgements, such that yellows were considered to shift categorically to green, and greens to blue. If so, this would be a particularly intriguing behaviour, as it would suggest that participants perhaps engaged semantic knowledge when perspective-taking but a different strategy when working alone. This would not explain the *full* pattern of results, because both groups showed bias on the yellowest tiles, and the No Perspective-Taking group also showed an anomalous shift away from “bluer” judgements on the final green tile. Nevertheless, we suggest it might be a fruitful avenue for future research to examine more directly the role of category labelling in perspective-taking and egocentric biases.

Our findings serve to underline an important question that is already being debated in the literature, namely the merits or otherwise of drawing conclusions about how we process others’ mental states when we are performing with real people or simulations of them. It has already been pointed out that we behave qualitatively differently towards “people” when faced with a depictions of them relative to when we are face-to-face with them (Skarratt et al., 2012). Indeed, part of the difficulty of situating our findings in the context of the wider literature might result from disparate behavioural patterns depending on the type of agent used in a task. More research with real human others as agents might be better placed to inform the study of social reasoning in our species.

## Conclusion

Taken together, we interpret the results as evidence that adopting a perspectival strategy when judging object appearance might reduce egocentric biases relative to a functionally equivalent but nonsocial rule, at least under conditions of high uncertainty.

## Supplemental Material

QJE-STD-19-212.R2-Supplementary_Material – Supplemental material for Reduced egocentric bias when perspective-taking compared with working from rules Supplemental material, QJE-STD-19-212.R2-Supplementary_Material for Reduced egocentric bias when perspective-taking compared with working from rules by Steven Samuel, Anna Frohnwieser, Robert Lurz and Nicola S Clayton in Quarterly Journal of Experimental Psychology

## Notes

1. Given that the colour filter was not computer-generated, the stimuli as seen through the filter did not correspond perfectly to the colours on the scale, hence the instruction to indicate which colour “best represents what the experimenter saw” or “best represents what you saw.”

2. We changed the group names in this experiment from Self-Judgement and Other-Judgement to No-Perspective-Taking and Perspective-Taking because participants in the No Perspective-Taking group did not see the target object half the time, and hence were not always judging something they themselves could see.

3. One participant’s data were later excluded as it was evident they were consistently reporting the true colour and ignoring the effect of the filter. Including their data did not alter the pattern of results.
