# Supplementary material for: Reduced egocentric bias when perspective-taking compared with working from rules
Source: Q J Exp Psychol (Hove). 2020 May 22;73(9):1368–81. doi: 10.1177/1747021820916707 (PMC7509608; doi:10.1177/1747021820916707)
Supplement: QJE-STD-19-212.R2-Supplementary_Material – Supplemental material for Reduced egocentric bias when perspective-taking compared with working from rules [file QJE-STD-19-212.R2-Supplementary_Material.docx]

Supplementary Material for:

**Reduced egocentric bias when perspective-taking compared to working from rules.**

Samuel Steven, Frohnwieser Anna, Lurz Robert, and Clayton Nicola S.

|  |  | True colour RGB (unfiltered) | | |  |  |
| --- | --- | --- | --- | --- | --- | --- |
| Scale tile | Stimulus | R | G | B | Scale location (y axis) | Colour |
| 1 | 1 | 255 | 242 | 0 | 25-41 | Yellow |
| 2 | 2 | 232 | 238 | 9 | 43-59 |  |
| 3 | 3 | 209 | 234 | 18 | 61-77 |  |
| 4 | 4 | 185 | 229 | 27 | 79-95 |  |
| 5 | 5 | 162 | 225 | 36 | 97-113 |  |
| 6 | 6 | 139 | 221 | 45 | 115-131 |  |
| 7 | 7 | 116 | 217 | 55 | 133-149 |  |
| 8 | 8 | 93 | 213 | 64 | 151-167 |  |
| 9 | 9 | 70 | 209 | 73 | 169-185 |  |
| 10 | 10 | 46 | 204 | 82 | 187-203 |  |
| 11 | 11 | 23 | 200 | 91 | 205-221 |  |
| 12 | 12 | 0 | 196 | 100 | 223-239 | Green |
| 13 | Scale only | 0 | 188 | 109 | 241-257 |  |
| 14 | Scale only | 0 | 184 | 117 | 259-275 |  |
| 15 | Scale only | 0 | 180 | 125 | 277-293 |  |
| 16 | Scale only | 0 | 175 | 134 | 295-311 |  |
| 17 | Scale only | 0 | 171 | 142 | 313-329 |  |
| 18 | Scale only | 0 | 167 | 150 | 331-347 |  |
| 19 | Scale only | 0 | 163 | 158 | 349-365 |  |
| 20 | Scale only | 0 | 159 | 166 | 367-383 |  |
| 21 | Scale only | 0 | 155 | 174 | 385-401 |  |
| 22 | Scale only | 0 | 150 | 183 | 403-419 |  |
| 23 | Scale only | 0 | 146 | 191 | 421-437 |  |
| 24 | Scale only | 0 | 142 | 199 | 439-455 | Blue |
